# Cancer, you fascist mass

**DOI:** 10.1093/oncolo/oyaf098

**Published:** 2025-05-11

**Authors:** Nicholas C DeVito

**Affiliations:** Duke University, Durham, NC, United States

Cancer, you fascist mass

A selfish thief of time

Joy

And freedom from worry

You take and destroy

What the body took so long

To create

Corrupting our defenses

Invading where you do not belong

Using functions you do not understand

All

For the sake of your growth

And in spite of what we built

Or who we love

Or our potential

One day, we will reject you

And heal

## Research Support:

No

Not presented elsewhere

No disclaimers

